# An investigation of cortical neuroplasticity following stroke in adults: is there evidence for a critical window for rehabilitation?

**DOI:** 10.1186/s12883-015-0356-7

**Published:** 2015-07-11

**Authors:** Michelle N. McDonnell, Simon Koblar, Nick S. Ward, John C. Rothwell, Brenton Hordacre, Michael C. Ridding

**Affiliations:** Alliance for Research in Exercise, Nutrition and Activity and International Centre for Allied Health Evidence, Sansom Institute for Health Research, School of Health Sciences, University of South Australia, GPO Box 2471, Adelaide, SA 5001 Australia; Stroke Research Programme, School of Medicine, South Australian Health and Medical Research Institute, University of Adelaide, Adelaide, Australia; Sobell Department of Motor Neuroscience, University College London Institute of Neurology, Queen Square, London, England; UCLP Centre For Neurorehabilitation, Queen Square, London, England; The National Hospital for Neurology and Neurosurgery, Queen Square, London, England; Neuromotor Plasticity and Development Research Group, Robinson Research Institute, School of Paediatrics and Reproductive Health, University of Adelaide, Adelaide, Australia

**Keywords:** Stroke, Neuroplasticity, Critical window

## Abstract

**Background:**

Evidence in animal stroke models suggests that neuroplasticity takes place maximally in a specific time window after an ischaemic lesion, which may coincide with the optimal time to intervene with rehabilitation. The aim of this study is to investigate neurophysiological evidence for a “critical window” of enhanced neuroplasticity in patients following ischaemic stroke, and establish its duration. We will also investigate changes in cortical inhibition following stroke, and the influence this has on functional recovery.

**Methods/Design:**

We will recruit participants recently admitted to the Stroke Unit of major metropolitan hospitals who have had a stroke and can provide informed consent. Participants will be excluded if they have any contraindications to Transcranial Magnetic Stimulation. We will compare neurophysiological outcomes in an age-matched healthy control group. We conservatively hypothesise a 5 % increase in neuroplasticity at the optimal timing following stroke, compared to control participants, and require 43 patients following stroke to detect a significant difference with 80 % power. The primary outcome is the change in the motor evoked potential (MEP) amplitude in a hand muscle, after the administration of a plasticity-inducing paradigm to the affected hemisphere. Secondary outcomes include measures of cortical excitability, intracortical inhibition and arm function.

**Discussion:**

The data from this trial will clarify whether there is a critical window for neuroplastic change in the brain following stroke. If so, intensive rehabilitation during this period could be more effective, reducing long-term disability and the cost burden of stroke.

## Background

Following stroke, a number of structural changes take place in the cortex that facilitate true recovery (restitution) of function. For example, there is evidence for a short period of new axonal growth in areas surrounding a cortical ischaemic lesion [1]. This allows for some active replacement of connectivity that has been damaged by the stroke. Maximising this neuroplasticity is likely to be an important avenue for optimising functional recovery.

There is overwhelming evidence that neuroplastic mechanisms are available throughout life and play an important part in stroke recovery [2]. However, there are also ‘critical periods’ during development in which neuroplastic capabilities are greatest and environmental/behavioural experience during these times results in major changes in structure and connectivity within the brain [3].

Direct evidence of such ‘critical period’-like neuroplasticity following stroke is limited in humans. However, neuroimaging and non-invasive stimulation techniques have provided important clues that such an enhanced period of neuroplasticity might be present [4–6]. It might be expected that rehabilitative interventions applied during this period would yield the best functional outcomes.

There are additional changes in the post-stroke brain that might affect neuroplasticity and influence recovery. Hyper-excitability occurs in the area surrounding a cortical lesion and is associated with greater long-term potentiation and reduced gamma-aminobutyric acid (GABA) mediated inhibition [7, 8]. Human data also suggests that GABAergic inhibition is reduced in the stroke affected hemisphere [9, 10]. Modulations in inhibition might influence functional neuroplasticity in the post-stroke brain.

Some behavioural data in humans suggest that there might be optimal windows for rehabilitation post stroke; the earlier that patients are admitted to stroke rehabilitation, the greater the functional gains [11]. In order to optimise the timing of post-stroke therapy, it is critical to determine the time-course of neuroplastic change within the stroke-damaged cortex.

## Methods/Design

### Objective

The primary aim of this study is to provide neurophysiological evidence of a critical window of enhanced neuroplasticity following stroke, and to determine whether the rate of functional recovery is greatest during the period of enhanced neuroplasticity. We anticipate that recovery may be linked to changes in cortical inhibition following stroke, and we will investigate this as a secondary aim of the study.

### Design

This study is a prospective cohort study of adults following a first-ever stroke. We will recruit participants from metropolitan hospitals in Adelaide, Australia and London, England.

### Patient population

We will recruit adults over the age of 18 with no upper age limit into the study. The detailed study inclusion and exclusion criteria are outlined in Table 1.

**Table 1:** Study inclusion and exclusion criteria

Inclusion criteria
Experienced a middle cerebral artery stroke with cortical involvement; either a first-ever stroke or at least 1 year after a stroke in non-motor regions
Medically stable
Confirmed diagnosis of stroke from CT/MRI imaging
Have mild/moderate hand weakness (defined in this study as being able to lift and hold a small object, but do not full strength according to Medical Research Council grades) [19]
Have recordable motor evoked potentials (>200 μV) to transcranial magnetic stimulation (TMS) in affected hand muscles
Exclusion criteria
Contraindications to TMS [20]
For example, history of other neurological disease, including epilepsy, cardiac pacemaker, metal implants in the skull. A complete TMS Safety Screening Questionnaire will be completed in accordance with international guidelines [20]
Severe receptive aphasia
Pre-morbid dementia
Inability to give informed consent

We will also recruit 35 age- and sex-matched healthy control participants to take part in the neurophysiological measures described below on three occasions, separated by six months. They will be included if they have no history of neurological disorders, and excluded if they have any contraindications to TMS. All participants will provide written, informed consent in accordance with the Declaration of Helsinki. The study procedures have been approved by the relevant Human Research Ethics Committees.

### Study procedures

All participants will receive usual care for stroke rehabilitation. Where this involves transfer to inpatient rehabilitation, we will provide transport for participants to return to the laboratory for neurophysiological assessments. In the event that participants are discharged home with residual arm weakness, a physiotherapist will prescribe and supervise a home exercise program based on the GRASP protocol (Graded Repetitive Arm Supplementary Program) [12].

**Fig. 1:**
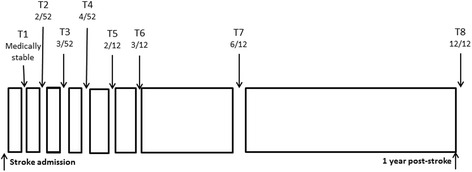
Timeline of assessments

Neurophysiological and functional assessments will be conducted up to eight times over the first year post stroke, as shown in Fig. 1. We anticipate that changes in neuroplasticity will be most evident in the first month post stroke, therefore this will be the period of most intense investigation.

### Primary outcome

We will examine a number of different neurophysiological measures, outlined below, to describe the presence of a critical window. The primary outcome of interest is the change in the motor evoked potential (MEP) amplitude in the hand, after the administration of a plasticity-inducing paradigm to the affected motor cortex. The amplitude of the MEP at any given stimulus intensity reflects the excitability of cortical synapses in the networks generating the MEP. We will use an inhibitory, spaced, continuous theta burst stimulation paradigm (cTBS) to induce short-term plasticity within the motor cortex of the affected hemisphere [13]. We will compare the amplitude of MEPs recorded at baseline, to those recorded at 5, 15, 30 and 45 mins following paired cTBS. The change in MEP amplitude in the affected hemisphere following stroke will be compared to that observed in the dominant hemisphere of age-matched healthy control participants.

### Secondary outcomes

Stimulus–response curves: we will use three stimulus intensities (110, 130 and 150 % resting motor threshold, RMT) to quantify the stimulus–response relationship over time.

*Motor threshold:* we will record resting and active motor threshold in the first dorsal interosseous muscle of the stroke-affected hand using standard techniques. This provides a measure of excitability in the cortical network activated by TMS (see [14] for review).

*Short interval intracortical inhibition (SICI):* we will use standard techniques [15] to record the motor cortical intracortical inhibition that occurs when a sub-threshold conditioning stimulus (70 % RMT) precedes a supra-threshold test stimulus (120 % RMT), at 2 and 3 ms inter-stimulus intervals.

We will also perform functional testing to assess rates of recovery of arm function following stroke. These include:

- National Institutes of Health Stroke Scale (NIHSS), a measure of stroke severity, recorded on admission to hospital.
- Functional Independence Measure (FIM), a measure of independence with mobility and activities of daily living.
- The Action Research Arm Test (ARAT), to measure arm activity in the domains pinch, grip, grasp and gross arm movement.
- The Grip-lift task, a sensitive indicator of dextrous hand function following stroke [16].

Finally, we will record the amount of arm movement performed by the affected arm using tri-axial accelerometers (Actiwatches) [17]. These will be worn on both arms for three consecutive days between assessments time points, and will be included in the analysis as a covariate.

### Sample size estimate

Our conservative estimate is for a 5 % increase in the neuroplastic response to cTBS at the optimal timing (i.e. during the *critical window*) following stroke. Considering the mean response and variance of data from control subjects to the paired cTBS paradigms [13] we would need approximately 43 patients to detect a difference (p < 0.05) at 80 % power. Therefore, to be confident of examining the hypotheses and allowing for a 33 % loss to follow up rate, we will recruit 70 patients over the four years of the grant.

### Statistical analyses

To address our aims we will perform the following analyses:

1. Repeated measures analysis of variance (rmANOVA) to examine changes across time in neuroplasticity (MEP response to cTBS) and inhibition (SICI).
2. ANOVA to compare neuroplasticity and inhibition between patients and controls over time.
3. Generalised Estimating Equations to examine the longitudinal relationship between neuroplasticity, intracortical inhibition, and functional assessment scores.
4. ANOVA comparing neuroplastic responses between patients stratified into tertiles based upon their functional assessment scores (ARAT/FIM) at the end of the trial period (poor recovery/moderate recovery/good recovery).

Where appropriate, lesion size/site (using assessment of corticospinal tract structure from automated segmentation of structural MRI) and upper limb activity scores will be used as covariates in the above analyses.

### Study organisation and funding

This study has been funded by the National Health and Medical Research Council (NHMRC) of Australia, Project Grant ID 1058639, from 2014–2017 and partially funded by a Medical Research Council (UK) Grant MR/K01384X/1 to JCR.

## Discussion

Little is known about the time course of neuroplastic potential in the damaged hemisphere following stroke, nor whether this impacts upon the effectiveness of rehabilitative strategies and true restitution of function. We will carefully examine changes in the corticospinal pathway, with MEP amplitude and stimulus response curves, in individuals from approximately one week to one year post stroke. We will also describe changes in intracortical inhibition, thought to influence functional recovery in the brain post stroke [18]. Most importantly, we will perform serial investigations of the response of the affected motor cortex to paired cTBS, designed to induce short-term plasticity in the motor cortex. Comparing this response to aged-matched controls, as well as longitudinal assessment of functional recovery, will provide definitive evidence, or lack thereof, for a critical window of neuroplasticity following stroke.

### Summary and conclusions

This study will further our understanding of the changes that occur in the damaged brain that support functional recovery following stroke. Optimising rehabilitation to a proposed critical window of neuroplasticity could help to improve both speed and the amount of recovery following stroke and could lead to significantly greater improvements in functional outcomes.

## Abbreviations

ARAT: Action Research Arm Test
FIM: Functional Independence Measure
GABA: Gamma-aminobutyric acid
GRASP: Graded Repetitive Arm Supplementary Program
MEP: Motor evoked potential
MRI: Magnetic resonance imaging
NIHSS: National Institutes of Health Stroke Scale
rmANOVA: Repeated measures analysis of variance
RMT: Resting motor threshold
SICI: Short-interval intracortical inhibition
TBS: Theta burst stimulation
TMS: Transcranial Magnetic Stimulation
